# Supplementary material for: High proton conductivity through angstrom-porous titania
Source: Nat Commun. 2024 Dec 4;15:10546. doi: 10.1038/s41467-024-54544-z (PMC11615327; doi:10.1038/s41467-024-54544-z)
Supplement: Supplementary file 1 — Supplementary Information [file 41467_2024_54544_MOESM1_ESM.pdf]

Supplementary Information

High proton conductivity through angstrom-porous titania

Yu Ji, Guang-Ping Hao, Yong-Tao Tan, Wenqi Xiong, Yu Liu, Wenzhe Zhou, Dai-Ming Tang, Renzhi Ma, Shengjun Yuan, Takayoshi Sasaki, Marcelo Lozada-Hidalgo, Andre K. Geim, and Pengzhan Sun

This file contains

Supplementary Notes (1-7)

1. HRTEM imaging
2. Helium leak tests
3. Density functional theory calculations
4. Thermal stability
5. High-temperature measurements
6. Zeta potential
7. Comparison with other proton-conducting pores

Supplementary Figures (S1-S9)

Figure S1. Optical images for titania monolayers placed on an oxidized silicon substrate.
Figure S2. Device fabrication.
Figure S3. HRTEM image over a larger area.
Figure S4. Helium leak measurements.
Figure S5. Simulations of proton permeation.
Figure S6. Stability of monolayer titania at elevated temperatures.
Figure S7. High-temperature measurements for a second device.
Figure S8. Drift-diffusion measurements.
Figure S9. Zeta potential.

Supplementary References (1-14)

1. HRTEM imaging

The extensive imaging shown in Fig. S3 (over 500 nm^2 in size) allows for a comprehensive statistic for the occurrence frequency of vacancies in the titania monolayers. To that end, we selected 6 areas in Fig. S3, which are of better contrast and each about 25 nm^2 in size. Then we estimated the density of vacancies by counting the number of blurred squares with respect to those with clear and dark centres. As discussed in the main text and in the previous report[1], these two features correspond to single-Ti-atom vacancies and intact lattices, respectively. The number density of vacancies in each area is shown in Fig. S3 and a combined area of over 150 nm^2 yields an occurrence frequency of (7.5 ± 0.4)%, in agreement with the previous work[1]. This frequency translates into an areal density of $\sim 10^{14}$ cm^{-2} or about one vacancy per nm^2.

2. Helium leak tests

Most of our tested devices (20 in total) exhibited no helium flows within the described sensitivity. The leak rates found at different pressures up to 1 bar were indistinguishable from those using bare silicon wafers (Fig. S4). On the other hand, two devices showed leak rates well above the detection limit. Figure S4 shows one of such examples. The leak rates increased linearly with the pressure and reached the order of 10^{13} atoms s^{-1} at a high feed pressure of 1 bar. The linear pressure dependence translates into a helium permeability of $\sim 10^8$ atom s^{-1} Pa^{-1} and assuming the Knudsen flow, this permeability corresponds to a pinhole of about 50 nm in size. Indeed, our retrospective inspection under an electron microscope (inset of Fig. S4) corroborated the presence of such a pinhole. The leaky devices were excluded from further measurements.

3. Density functional theory calculations

The simulation results are shown in Figs. S5b, c. For both intact lattice and vacancy, the energy profiles for the permeation of protons are attractive due to the negative charges from the surface O atoms, resulting in a deep energy well with two energy minima. The energy minima correspond to the most stable adsorption configurations of protons to the oxygen atoms on both sides of the crystal. The surface adsorption states can then be considered as the initial states prior to translocation. This is in accordance with our measurements shown in Fig. 4 of the main text, which emphasize the importance of surface adsorption to the observed proton transport. With protons crossing through the crystal, an energy maximum (transition state) emerges due to electrostatic repulsion from the surrounding Ti atoms and the permeation barrier can be estimated as the energy step between the initial and transition states. From the energy evolution of a proton piercing an intact titania lattice, the calculated barrier height is ~1.2 eV (Fig. S5b). This value is notably higher than our experimental activation energy (0.34 ± 0.06 eV) and also higher than those measured for graphene (~1 eV) and monolayer hBN (~0.5 eV)[2,3]. In contrast, the presence of a single-Ti-atom vacancy significantly lowers the barrier down to about 0.4 eV (Fig. S5c), which is in reasonable agreement with our measurements. The described trend in E is also in qualitative agreement with our calculations for the electron densities (Fig. S5a), which show that the electron clouds are notably sparser at vacancy sites than in either hBN, graphene or intact-titania monolayers. Specially, the energy barrier observed in calculations (Fig. S5c) is slightly higher than that in experiments (Figs. 2c and 3). We tentatively attribute it to the presence of nanoscale corrugations in monolayer titania membranes. Suspended 2D membranes are practically not flat but subjected to nanoscale rippling due to

thermal fluctuations and local strain[4-7]. According to the recent reports[8,9], these nanoripples can lower the barrier for proton transport in graphene, hBN and graphene oxide. We believe the same could happen in monolayer titania. Comparing with the intact lattice, the energy profile calculated for a single vacancy exhibits a small asymmetry with respect to the transition state (Fig. S5c). This asymmetry can be attributed to the asymmetric structure of the created vacancy (insets of Fig. S5c), which results in different adsorption energies of protons to the opposite entrances of the vacancy. However, the asymmetry observed in the calculation is for a given vacancy site whereas in practice, high-density vacancies are expected to evenly distribute on both surfaces in the experimental sample, leading to symmetric and linear *I-V* responses in all our measurements.

Furthermore, we note that in an aqueous atmosphere, the strong negative surface charge can be, however, compensated by the mobile and positively charged species in water, for example, protons (Fig. S9, see below). This should make the surface less charged and therefore, result in a shallower energy well than that observed in our simulations (Fig. S5). Because of this complexity associated with the practical measurement environment, the above simplified model can only serve as a qualitative estimate. Nonetheless, it is still informative in allowing us to understand the important role of vacancies as identified in HRTEM images (Figs. 1c and S3) for the observed proton transport.

Finally, note that our isotope effect measurements yielded $\sigma_H/\sigma_D = 1.6 \pm 0.16$, which is smaller than $\sigma_H/\sigma_D \approx 10$ observed previously for proton transport through graphene and hBN monolayers[3]. The latter ratio was attributed to the energy difference $\Delta E \approx 60$ meV for zero-point oscillations of H^+ and D^+ that were bound to oxygen atoms before translocating through the 2D crystals. Along the same lines, using the expression $\sigma_H/\sigma_D \sim \exp(\Delta E/k_B T)$, the observed ratio of ~1.6 translates into a 12 meV difference in zero-point energies of H^+ and D^+. This suggests a notably shallower binding potential for protons and deuterons in their initial state bound to the titania surface, consistent with the analysis described above. On the other hand, our simulations that included quantum (zero-point) corrections showed that the initial surface binding states prior to translocation (that is, the energy minima in Fig. S5c) are slightly different in energy. This results in a barrier that is ~12 meV higher for D^+ than H^+, in good agreement with the experiment.

4. Thermal stability

As shown in Figs. S6a and b, we found no changes in the shape and intensity of characteristic peaks assigned to Ti $2p_{1/2}$, Ti $2p_{3/2}$ and Ti-O bonds, after heating the samples to 300 °C in various gas atmospheres. The only change observed in the O 1s spectra was a notable reduction in the intensity of the peak due to hydroxyl groups (Fig. S6b), and this can be attributed to the loss of remnant water adsorbed on the crystal's surface rather than the latter's structural changes. Furthermore, the annealed crystals were carefully examined under AFM and Fig. S6c shows one of such images. The material still preserved its 2D structure after thermal treatment and no noticeable defects such as pinholes, cracks and tears were observed. These characterizations indicate that 2D titania exhibits good thermal stability which is intrinsic to bulk oxides and their chemical/crystallographic structures can be well-retained in the full range of our measurement temperature.

5. High-temperature measurements

At room *T*, the areal conductivity of protons σ measured with Pt-coated devices is lower than that for the Nafion-coated ones. This could be attributed to either some vacancies being blocked by the Pt atoms or

to a lower density of protons on the crystal surface. Nonetheless, both possibilities are rooted at a lower number of protons accessing the vacancies and can be corroborated by depositing Pt films of different thicknesses. To that end, in addition to the device shown in Fig. 3, we deposited denser Pt films (~40 nm thick) on both sides of a different device and measured its *T* dependences. As shown in Fig. S7, σ measured at room *T* was lowered further with respect to the device having ~10 nm thick Pt films (Fig. 3). The conclusion about a lower density of incident protons is also supported by practically the same activation energies of ~0.36 eV observed on both Nafion- and Pt-coated devices (Figs. 3 and S7), suggesting the same mechanism governing the observed proton transport. Despite the lower room-*T* areal conductivities, the Pt-coated devices could operate satisfactorily at elevated *T* extending into the proton materials gap (200 – 500 °C)[10] and the observed σ reached 100 S cm^{-2} at 200 °C (Fig. 3) and 200 S cm^{-2} at 260 °C (Fig. S7), much higher than the target (50 S cm^{-2}) set in the industry roadmap as discussed in the maintext. Notice that the Arrhenius plots for Pt-coated devices deviate slightly from the exponential fit at elevated *T* (Figs. 3 and S7). We suggest this could be related to the presence of adsorbed water on the surfaces of titania membranes. Such water layers are expected to facilitate proton injection from the Pt films to the titania surfaces. However, the equilibrium concentration of the adsorbed water layers is expected to decrease with increasing *T* (Fig. S6b), which could explain the slight increase in the activation energy of the proton transport process at higher *T*.

6. Zeta potential

Figure S9 plots our measured ζ data as a function of pH. In the higher pH regime (3–6), $\zeta \approx -55$ mV and exhibited little changes with pH. The large and negative ζ indicates a strong negative charge on the surface of titania monolayers. This is consistent with the fact that formation of single-Ti-atom vacancies leaves high-density negative charges on the crystal's surface. With further increasing the concentration of protons, we found ζ rapidly decreased to zero and then switched its polarity to positive at pH ≈ 1. These results clearly show the strong adsorption of protons to the surface of 2D titania – the adsorbed protons neutralize the crystal's negative charges and then excessive protons further positively charge its surface.

Assuming the slipping plane overlaps with the outer surface of Helmholtz layer, to a first approximation, the surface charge density ρ_s can be estimated from the measured zeta potential ζ according to the Gouy–Chapman theory of electric double layers[11]:

$$\rho_s = \varepsilon\varepsilon_0\zeta\kappa$$

Here, ε and ε_0 are the dielectric constant of water and the permittivity of free space, respectively, and κ^{-1} is the Debye length, given by $\kappa^{-1} = \sqrt{\frac{\varepsilon\varepsilon_0 k_B T}{2e^2 I}}$ with *e* the elementary charge and *I* the ionic strength (= *C*, for the HCl solution, in unit of number per m^3). This equation is valid in the limit of small eζ with respect to k_BT (≈ 26 meV at room *T*), and therefore, we can only use it to estimate ρ_s in the vicinity of pH = 1 where $|\zeta| < 26$ mV. The estimations are included in Fig. S9, showing that ρ_s changes from –10 mC m^{-2} to +17 mC m^{-2} with decreasing the pH from 1.5 to 1.

7. Comparison with other proton-conducting pores

It would be instructive to compare the conductivity per pore and the activation energy of our titania membranes with other well-documented biological and solid-state pores or channels[12-14]. From the

occurrence frequency of single vacancies in the titania crystal and the measured areal conductivities, the conductance per pore after normalization by its length is estimated to be of the order of 10^{-6}–10^{-5} nS/nm. One key solid-state reference system is the carbon nanotube porins[12], with diameter of ~0.8 nm and length of ~10 nm. Their length-normalized conductance was of the order of 10^{-6} nS/nm and the activation energy was ~0.6 eV. Another widely studied biological reference system is the biological channel gramA[13,14], having a diameter of ~0.4 nm and about 5-nm long. The length-normalized conductance was ≈0.6×10^{-6} nS/nm and the activation energy was ≈0.2 eV. Despite the deviations observed in different systems, the length-normalized conductance values are all roughly of the order of 10^{-6} nS/nm within a factor of ~10 and the energy barriers are about 0.3 eV within a factor of ~2. This comparison implies that proton transport in 1D systems might display similar features across a wide range of systems, either biological or solid-state.

Supplementary figures

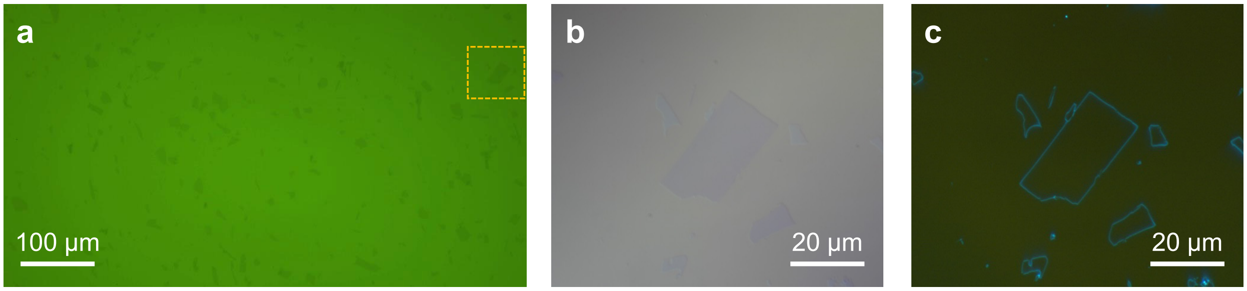

Figure S1 I Optical images for titania monolayers placed on an oxidized silicon substrate. a, Bright field image green filtered for better contrast. **b**, Differential interference contrast micrograph of the flake square marked in (a). **c**, Same flake as in (b) but under the dark field mode.

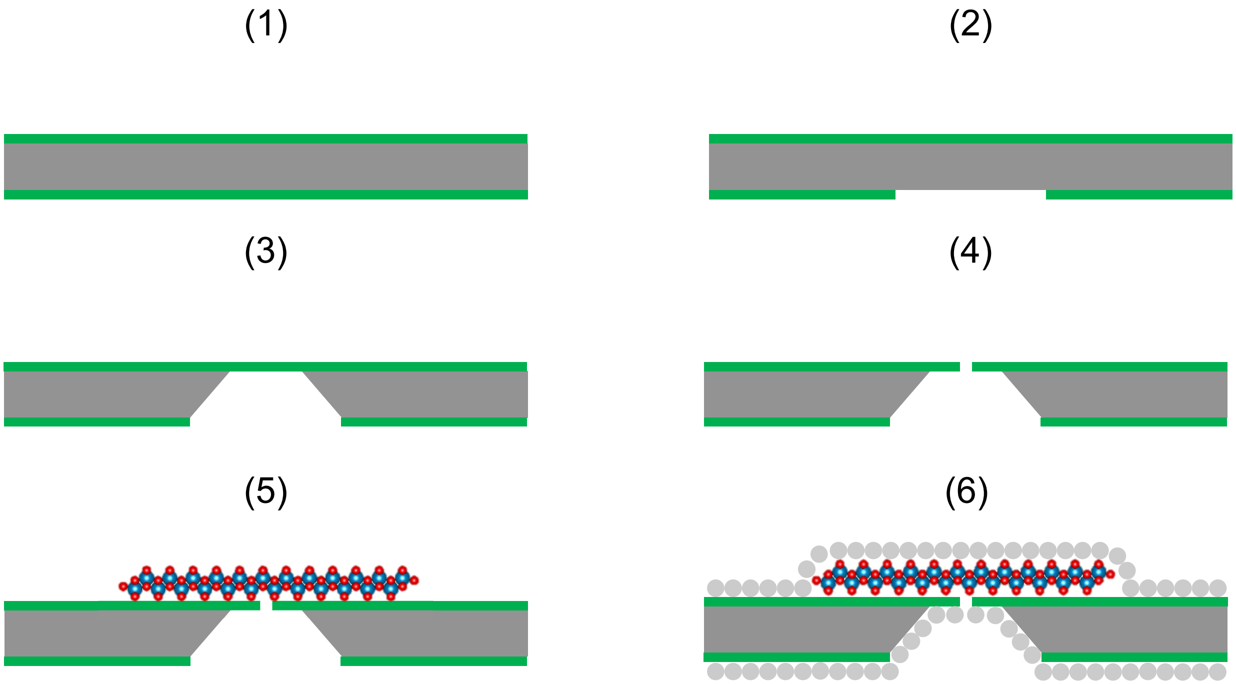

Figure S2 I Device fabrication. (1) A square of 800 µm × 800 µm in size was defined using photolithography on a silicon wafer which was coated on both sides with 500 nm thick SiNx. (2) Then dry etching was employed to completely remove the uncovered SiNx so that silicon was exposed. (3) After etching through the exposed silicon using concentrated KOH solution at 90 ^{o}C, a piece of freestanding SiNx membrane was produced. (4) Another round of photolithography and dry etching was employed to fabricate an aperture 2-3 µm in diameter through the freestanding SiNx membrane. (5) Finally, the selected 2D crystal such as the one in Fig. S1 was transferred over the aperture. (6) For higher temperature measurements, porous Pt films were sputtered on both sides of the fabricated device in (5).

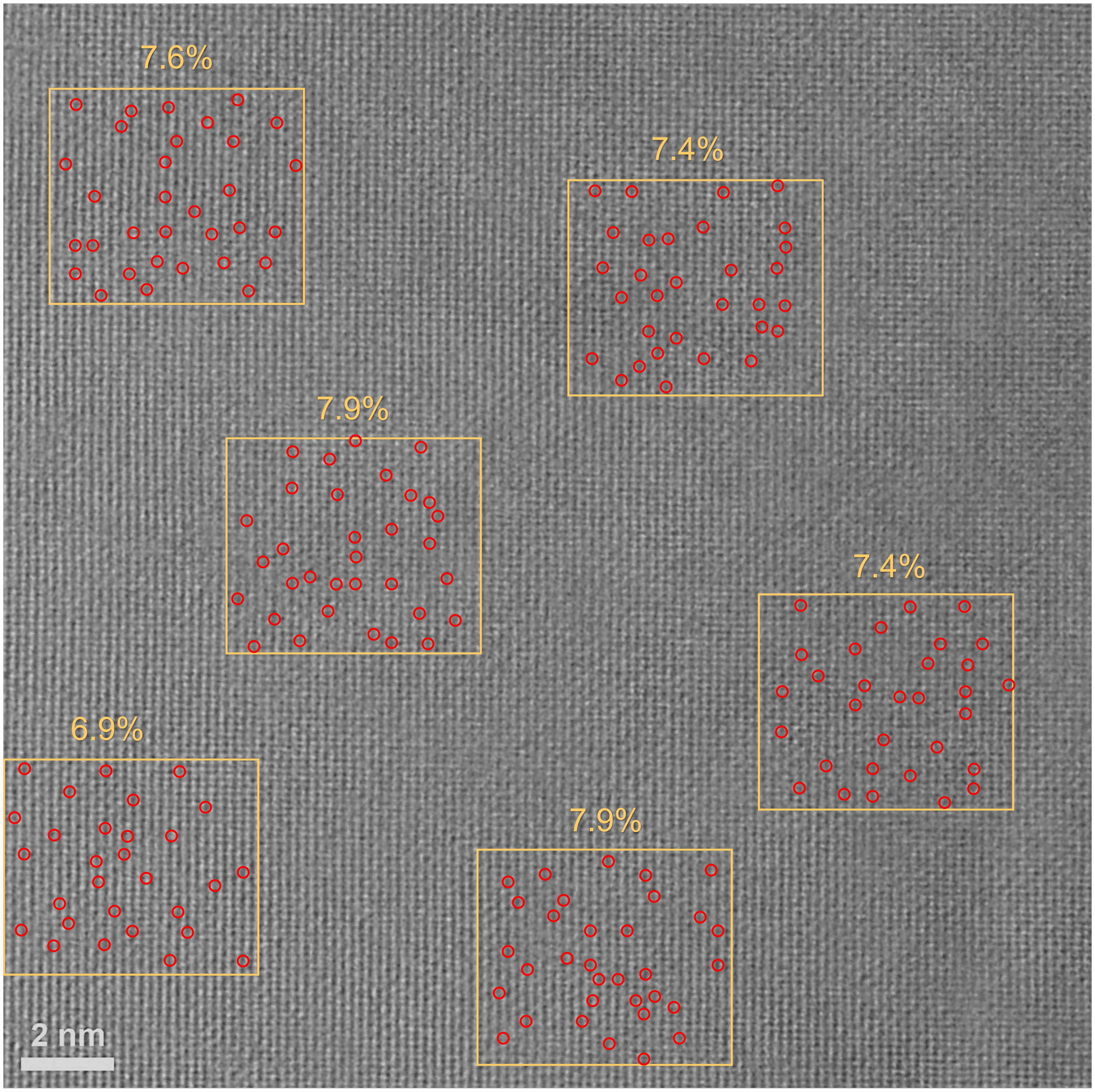

Figure S3 I HRTEM image over a larger area. The yellow squares mark regions where statistics analyses for the density of vacancies (red circles) were performed with its number shown above.

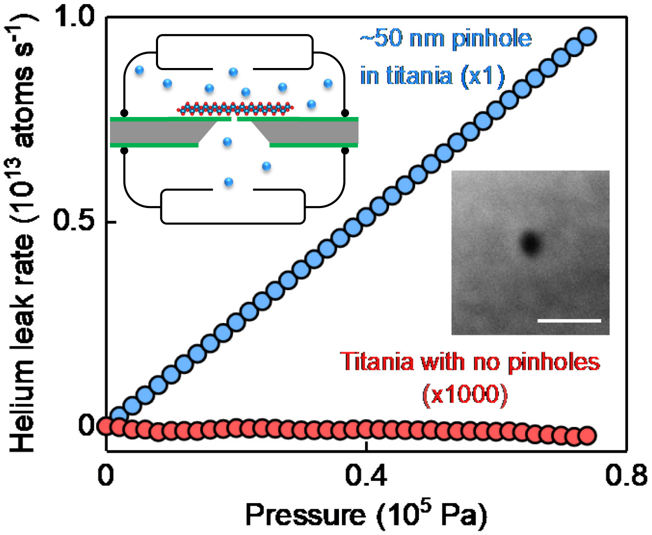

Figure S4 I Helium leak measurements. Shown are helium leak rates versus pressure for a leaky titania device and an impermeable one (colour coded). The latter's signals are amplified 1,000 times. Top inset, measurement setup. Bottom, electron micrograph showing a ~50 nm size pinhole found in the leaky device.

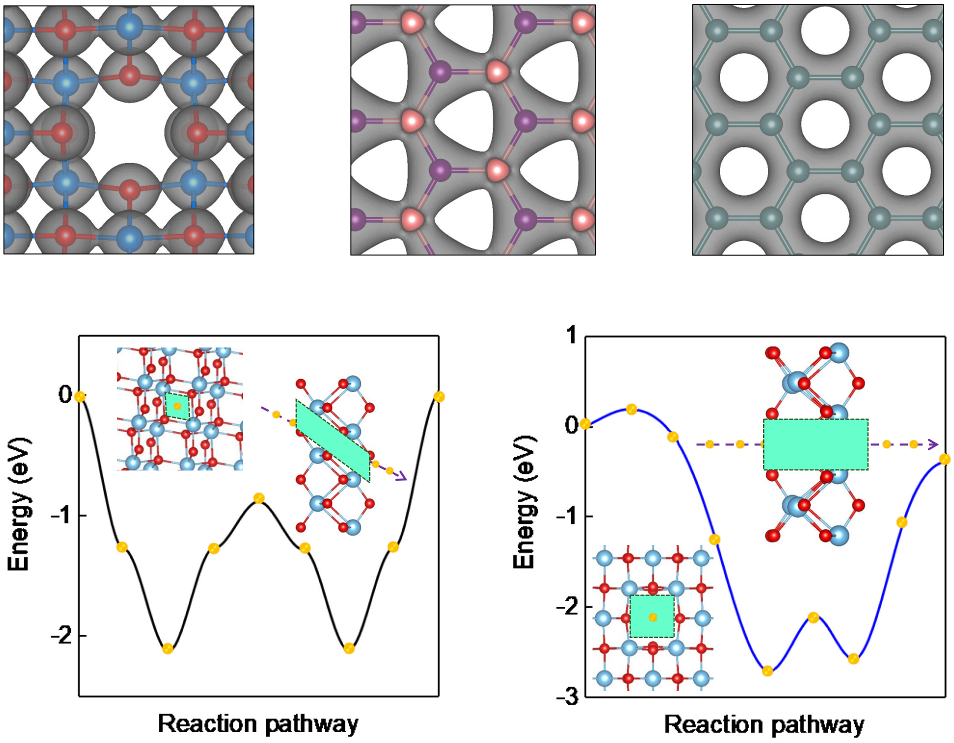

Figure S5 I Simulations of proton permeation. a, Calculated electronic densities for 2D titania with vacancies, hBN and graphene (from left to right). Grey areas: isosurfaces at 0.6 e/Å^3. **b** and **c,** Energy

profiles for translocation of a proton through a pristine titania lattice and a single Ti-atom vacancy, respectively. Insets, schematics for the top-view (left) and cross-sectional view (right). The green boxes outline the minimum-energy pathways for protons. The yellow symbols on the curves correspond to different states of the simulated proton as illustrated in the right insets (from left to right).

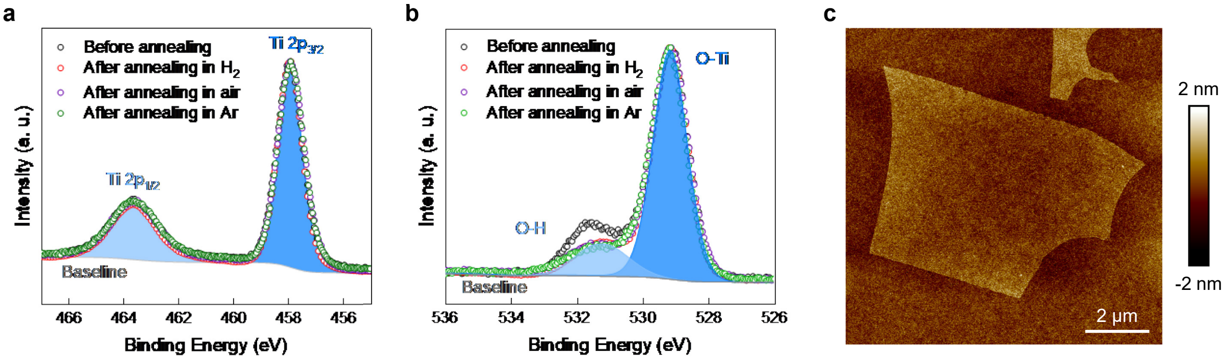

Figure S6 I Stability of monolayer titania at elevated temperatures. XPS spectra for titania monolayers before and after annealing in different gases (color coded) at 300 °C for 3 h. **a**, Ti 2p; **b**, O 1s. Symbols, experimental data. The shaded areas contour with blue curves and the best fits for the data taken before annealing; grey curves, baselines. **c**, AFM image of one of our flakes after annealing in air at 300 °C, showing its unaffected structure.

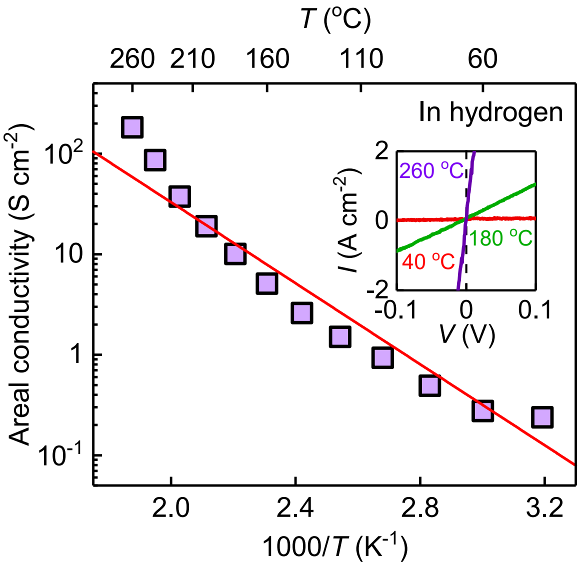

Figure S7 I High-temperature measurements for a second device. Denser Pt films of ~40 nm thick were deposited on both sides of the titania membrane. Purple symbols, experimental data. Solid line, guide to the eyes, showing the Arrhenius dependence with activation energy = 0.36 eV. Inset, representative *I*-*V* curves at different *T* (colour coded), from which the areal conductivities shown in the main panel were calculated. Black dashed line marks the zero voltage axis.

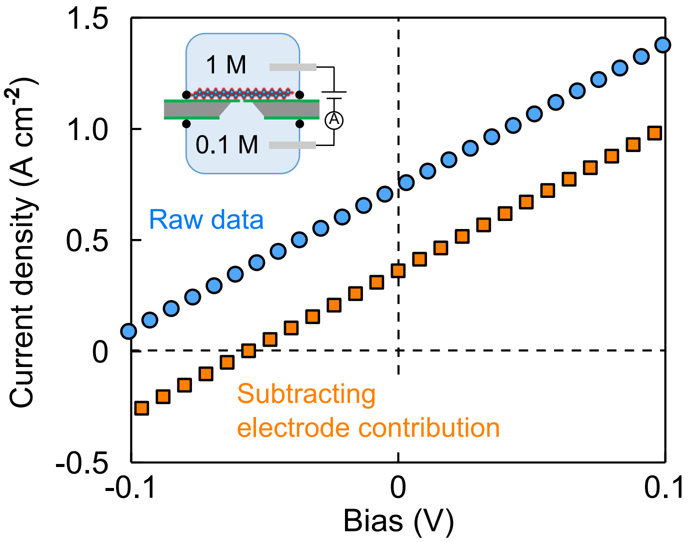

Figure S8 I Drift-diffusion measurements. Blue symbols: raw data including contributions from both selective ion transport and redox reactions at the electrodes. Orange symbols: same data after subtracting the volatge drop at the electrodes. The dashed lines indicate zero voltage and zero current. Inset, schematic of the experimental setup.

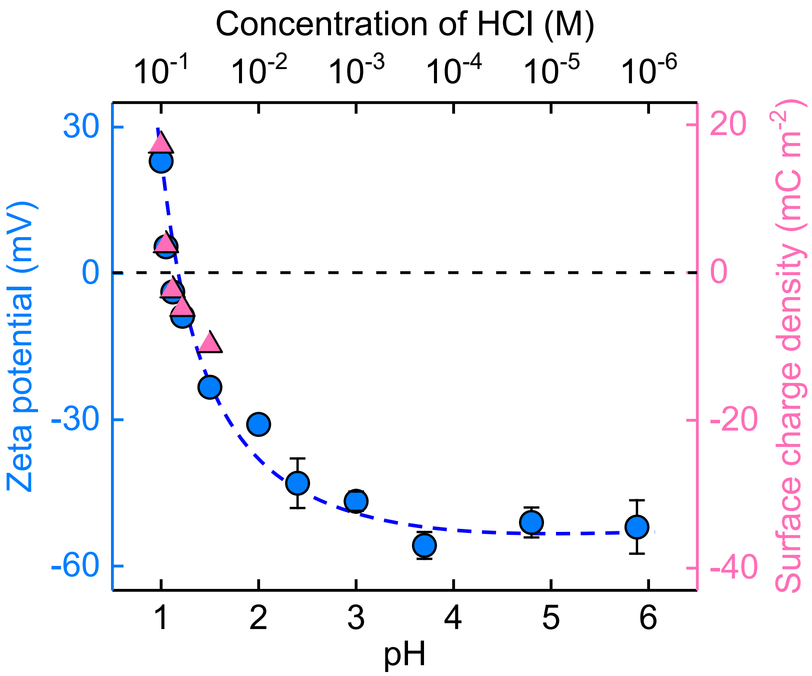

Figure S9 I Zeta potential. Titania crystals were delaminated in water and pH of the resulting dispersion was adjusted using HCl. Blue symbols, experimental data for the zeta potential measurements (left-y). Error bars, SD for three independent measurements (those smaller than the symbols are omitted). Pink symbols, surface charge densities estimated from the zeta potentials around pH = 1 (right-y). Blue dashed line, guide to the eyes. Black dashed line marks the position of zero ζ and zero ρ_s.

References

1. Ohwada, M., Kimoto, K., Mizoguchi, T., Ebina, Y. & Sasaki, T. Atomic structure of titania nanosheet with vacancies. *Sci. Rep.* **3**, 2801 (2013).
2. Hu, S. et al. Proton transport through one-atom-thick crystals. *Nature* **516**, 227–230 (2014).
3. Lozada-Hidalgo, M. et al. Sieving hydrogen isotopes through two-dimensional crystals. *Science* **351**, 68-70 (2016).
4. Meyer, J. C. et al. On the roughness of single- and bi-layer graphene membranes. *Solid State Commun.* **143**, 101–109 (2007).
5. Fasolino, A., Los, J. H. & Katsnelson, M. I. Intrinsic ripples in graphene. *Nat. Mater.* **6**, 858–861 (2007).
6. Zan, R. et al. Scanning tunnelling microscopy of suspended graphene. *Nanoscale* **4**, 3065–3068 (2012).
7. Xu, P. et al. Unusual ultra-low-frequency fluctuations in freestanding graphene. *Nat. Commun.* **5**, 3720 (2014).
8. Wahab, O. J. et al. Proton transport through nanoscale corrugations in two-dimensional crystals. *Nature* **620**, 782-786 (2023).
9. Wu, Z. F. et al. Proton and molecular permeation through the basal plane of monolayer graphene oxide. *Nat. Commun.* **14**, 7756 (2023).
10. Norby, T. Solid-state protonic conductors: principles, properties, progress and prospects. *Solid State Ionics* **125**, 1–11 (1999).
11. Rieger, P. H. Electrochemistry. Springer Netherlands (1993).
12. Tunuguntla, R. *et al.* Ultrafast proton transport in sub-1-nm diameter carbon nanotube porins. *Nat. Nanotechnol.* **11**, 639–644 (2016).
13. Gensure, R. H., Zeidel, M. L. & Hill, W. G. Lipid raft components cholesterol and sphingomyelin increase H^+/OH^- permeability of phosphatidylcholine membranes. *Biochem. J.* **398**, 485–495 (2006).
14. Chernyshev, A. & Cukierman, S. Thermodynamic view of activation energies of proton transfer in various gramicidin A channels. *Biophys. J*. **82**, 182–192 (2002).
